# Blue-Green Algae as Stimulating and Attractive Feeding Substrates for a Mediterranean Commercial Sea Urchin Species, *Paracentrotus lividus*

**DOI:** 10.3390/life13071510

**Published:** 2023-07-05

**Authors:** Paolo Solari, Giorgia Sollai, Viviana Pasquini, Angelica Giglioli, Roberto Crnjar, Piero Addis

**Affiliations:** 1 Department of Biomedical Sciences, Section of Physiology, University of Cagliari, 09042 Monserrato, Italy; gsollai@unica.it (G.S.); crnjar@unica.it (R.C.); 2 Department of Life and Environmental Sciences, University of Cagliari, 09126 Cagliari, Italy; viviana.pasquini@unica.it (V.P.); giglioli.angelica@gmail.com (A.G.); addisp@unica.it (P.A.)

**Keywords:** aquatic chemoreception, sea urchin, behavior, Klamath, spirulina, attractiveness

## Abstract

Sea urchins rely on chemical senses to localize suitable food resources, therefore representing model species for chemosensory studies. In the present study, we investigated the chemical sensitivity of the Mediterranean sea urchin *Paracentrotus lividus* to the blue-green alga *Aphanizomenon flos-aquae*, namely “Klamath”, and to a few amino acids chosen from the biochemical composition of the same algae. To this end, we used the “urchinogram” method, which estimates the movement rate of the sea urchins in response to chemicals. Our results showed that Klamath represents a strong chemical stimulus for *P. lividus* as it elicits an overall movement of spines, pedicellariae, and tube feet coupled, in some cases, to a coordinated locomotion of the animals. Sea urchins also displayed a sensitivity, even if to a lesser extent, to leucine, threonine, arginine, and proline, thus implying that the amino acids contained in Klamath may account, at least in part, for the stimulating effects exerted by the whole algae. Additionally, our results show that Klamath, as well as spirulina, another blue-green alga with high nutritional value, is very attractive for this sea urchin species. These findings gain further importance considering the potential profit of echinoderms for commercial consumers and their growing role in aquaculture. Klamath and spirulina combine high nutritional profiles with attractive and stimulating abilities and may be considered potential valuable feed supplements in sea urchin aquaculture.

## 1. Introduction

As slow-moving, broadcast spawning invertebrates, sea urchins heavily rely on waterborne chemical cues to produce adequate behavioral responses, ranging from spatial orientation to social communication, detection and avoidance of predators or damaged conspecifics, localization of suitable habitats, mates and, obviously, potential food resources [1]. It is known that sea urchins respond to algae recognized as food or other distant feeding stimuli using odor-guided rheotaxis even under turbulent water flow conditions [2,3,4,5]. The slow speed of these animals facilitates time-scale sampling of chemical stimuli, and the variety of chemosensory organs can improve spatial resolution [6]. Despite the broad chemical sensitivity exhibited by both larvae and adults, the chemoreceptive organs of sea urchins are still uncertain. Based on behavioral and histological studies, three main systems are believed to respond to chemical cues: the “spine system”, the tube feet, and the pedicellariae. The responses range from a simple reflex to a fully coordinated chemotaxis where the sea urchin moves toward or away from a stimulus source [7,8]. The chemoreceptors of sea urchins belong to the family of G-protein-coupled receptors (GPCRs), and their large number (up to several hundred) and high complexity level are comparable with those described in many other animals [9,10].

Among the marine invertebrates used in bioassays for feeding behavior and chemosensitivity, we identify the echinoid *Paracentrotus lividus* (Lamark 1816), a sea urchin widespread in the Mediterranean and Atlantic Europe [11]. From an ecological point of view, this species is one of the most important grazers of marine benthic sublittoral communities [12,13] and, given the lack of natural predators, may strongly influence marine benthic communities, in some cases driving the transition from erect macroalgal assemblages to coralline barrens [14,15,16]. *P. lividus* is a species of commercial importance, with high market demand for its roe, particularly in Mediterranean countries where natural stocks have become seriously over-fished [17,18,19,20,21]. To solve this problem, some Mediterranean countries have experimented with restoking plans using reared juveniles from experimental hatcheries [22,23], demonstrating that “conservative” aquaculture could represent an option to fill the gap between supply and market demand, thus ensuring long-term sustainability. For these reasons, several studies have been developed to formulate suitable feeds to ensure faster adult growth and gonadal maturation [24,25]. In this regard, peripheral chemosensitivity plays an important role by allowing animals to collect useful information for suitable food choices [26,27,28,29,30,31]. Numerous efforts have been made to improve the palatability of prepared diets, capable of stimulating food intake by sea urchins and fostering their growth [32,33]. On the other hand, the need to find high-protein sustainable ingredients as a fishmeal alternative is a critical step in proceeding with feasible aquaculture [34]. In this context, different forms of the green seaweed *Ulva* (fresh, defrosted, and fragmented cultured waters) were recently found to be stimulating substrates for the sea urchin *P. lividus*, and the fresh preparation also showed a markedly attractive effect [35]. Cyanobacteria and other microalgae have emerged as potential candidates for a sustainable supply of food sources and secondary metabolites of nutritional, cosmetic, and medicinal importance [36]. *Arthrospira*, *Chlorella*, and *Aphanizomenon* are microalgal genera largely employed as food sources and food supplements. This is for their high content of essential nutrients and proteins coupled with several bioactive components like essential fatty acids, vitamins, minerals, and pigments with antioxidant properties [37,38,39,40,41]. Spirulina (*Arthrospira platensis*) has proved to be an effective integrator in the formulation of fish feeds [42,43], and some benefits, such as roe enhancement and gamete production, have also been reported for the sea urchin species *P. lividus* [44]. Coherently, this sea urchin was recently found to display marked chemosensitivity to spirulina [45], and this evidence suggests the need to carry out further research on the bioavailability of proper nutrients for the preparation of biologically and economically optimized feeds for aquaculture purposes.

In the present study, we aimed to evaluate the chemical sensitivity and the chemotaxis of the sea urchin *P. lividus* to the blue-green algae *Aphanizomenon flos-aquae*, a unicellular prokaryotic microorganism belonging to the cyanobacteria phylum growing in Upper Klamath Lake (Oregon, OR, USA) and now considered as a “superfood” for its rich nutritional profile [46]. We also tested the stimulating efficacy of a few amino acids selected among the most abundant (weight ratio) ones from the biochemical composition of the Klamath blue-green algae to ascertain if the stimulating effects of the latter may be attributable, at least in part, to its single components of the aminoacidic fraction. To perform this, we used a method that assessed the movements of spines, tube feet, pedicellariae, and finally, the entire individual as a response to chemicals, which has previously been employed on the same sea urchin species [45].

Moreover, since chemical sensitivity does not necessarily imply attractive or repulsive chemotaxis, as a second aim of this study, we evaluated the attractivity level of Klamath, as compared to that of *Arthrospira platensis*, another blue-green alga for which stimulating effectiveness on this sea urchin was previously detected [45].

## 2. Materials and Methods

### 2.1. Animal Collection and Rearing Conditions

All experiments were performed on specimens of *P. lividus* with 30 mm in test diameter (third age class), collected from the south coast of Sardinia (39°06′45.5″ N, 9°00′52.3″ E). Before experiments, they were acclimated for two weeks in Plexiglas^®^ tanks (100 cm long, 50 cm wide, and 20 cm high), containing about 60 L of natural, aerated seawater (SW) at 20 ± 0.5 °C, 34‰ salinity, with a 12 h light/12 h dark photoperiodic regime. Animals were fed ad libitum three times a week with the green macroalga *Ulva* sp.; uneaten food and/or fecal material were removed every 2 days by means of a partial (less than 10%) water exchange. All experiments were carried out in full accordance with the EU Directive 2010/63/EU.

### 2.2. Sea Urchin Bioassay

The sea urchins were singularly exposed to the stimuli in a plastic tank (12.5 cm long × 7 cm wide × 7 cm high) containing ca. 350 mL of seawater (SW), according to the procedure used by Solari et al. [45]. Two plastic tubes (40 cm in length, 0.4 cm in diameter) connected the two opposite short ends of the tank to a peristaltic pump (Gilson, Minipuls Evolution^®^) using two different channels, which acted as the inflow and outflow terminals for delivery and removal of the stimuli (flow rate of 10 mL/min; Figure S1). The outflow terminal was connected to a wastewater collection system, consisting of a plastic bottle placed below the experimental arena. The animals were preliminarily left to acclimatize until they became motionless, which typically occurred within 15 min. At the beginning of each experiment, the response of each sea urchin to a SW blank control was monitored for 5 min. 

Aliquots of the stimuli were then administered to the tank for 1 min by switching the inflow terminal from SW to a different stimulus-containing reservoir, and each animal was given 4 min to respond, which began from the time the stimulus reached the experimental tank (about 45 s after switching). Trials were recorded using a color digital camera (Samsung SMX-F34, Samsung, Republic of Korea) positioned over the experimental tank. The animal response was investigated by evaluating: (a) the movement rate of spines; (b) the movement of tube feet; and (c) the fully coordinated locomotion of the animal, if present.

### 2.3. Stimuli and Supply Protocol

The blue-green alga *Aphanizomenon flos-aquae* (hereafter referred to as Klamath) and some essential (leucine, lysine, and threonine) and non-essential (arginine and proline) amino acids chosen as the most abundant (weight ratio) ones from the Klamath biochemical composition were selected as stimuli. The amino acids, already known for their potential feeding significance for aquatic animals [47,48,49,50], were purchased from Sigma-Aldrich (Milan, Italy), while Klamath microalgae were obtained from ZenStore (Italy).

All amino acids were first dissolved in SW at 10^−1^ mol/L and stored frozen as stock solutions. On the day of the experiments, they were thawed and diluted in SW to be used at the three different increasing concentrations 10^−5^, 10^−3^, and 10^−1^ mol/L [45,51]. The finely hashed powder of Klamath microalgae was suspended in SW at 5 mg/mL and then diluted at 2, 1, 0.1, and 0.01 mg/mL. To avoid any mechanical stimulation for the sea urchin, any particulate was carefully removed.

At the beginning of the experiment, a blank stimulation with SW was administered for 5 min to every sea urchin, and then the animal was exposed to the three concentrations of a given amino acid (five in the case of Klamath). During this trial of the stimulation sequence, the water in the experimental tank was not replaced (stepwise stimulations were used [45,52]).

### 2.4. Detection of Sea Urchin Movements

Visible movements of the sea urchin spines and tube feet and the fully coordinated locomotory activity, if present, of the whole animal within the experimental tank, were video-recorded and evaluated by way of a frame-to-frame PC analysis [45,46,47,48,49,50,51,52,53]. This approach produces an “urchinogram” in which the movements at several sites and levels on the same animal can be recorded and compared. The video recordings were converted to a resolution of 640 × 480 pixels, at 5 frames/s (300 frames/min). In this way, each frame could account for the instantaneous “movement state” of the sea urchin at a 200 ms interval. Each video was analyzed by a custom program, which estimates the animal movements by using a number of lines overlaid on the video frames in order to cross the dark/light boundary between the dark animal and the clear background (Figure S2). To cover the whole area of the experimental tank, we used a grid with 22 (13 vertical and 9 horizontal) equidistant lines [35,45]. In other words, the movements of the spines, tube feet, and locomotion of the dark silhouette of the animal on the clear background generate variations in pixel intensity along the lines that can be considered as an index of the movement rate of the animal. The mean square difference in pixel intensity along the lines in the grid between successive pairs of frames was considered and plotted during the whole experiment as it provides great sensitivity and good discrimination of the movements.

### 2.5. Detection of Microalgae Attractiveness

Sea urchins were individually exposed to Klamath and/or spirulina microalgae in circular plastic tanks (30 cm in diameter, 8 cm high), each containing about 4 L of SW (20 ± 0.5 °C and 34‰ salinity), according to the procedure already used by Addis et al. [35]. Animals were starved for 48 h preceding the experiments. The microalgae were supplied to the animals using the polyvinyl chloride (PVC) dispenser technique [54]. The dispensers were prepared by coating a rigid plastic rod (8 cm long × 0.5 cm wide) with a mixture containing the finely hashed powder of the microalgae suspended in PVC (code 389293, Sigma-Aldrich, Milan, Italy). The PVC substrate was preliminarily prepared by dissolving the PVC powder (125 mg/10 mL) in dichloromethane (CH_2_Cl_2_) and agitating it at 60–70 °C until complete solubilization, which typically occurred in a few minutes. The microalgae were then suspended at 5 mg/mL in the PVC substrate when the latter was still liquid; 2 aliquots of 500 µL of the PVC/attractant mixture were then poured on the opposite ends of the plastic rod and left to cool until solidification, typically 4–5 min. In this way, the microalgae powder remained trapped within the solid PVC matrix adhering to the rod, which could be easily supplied to the sea urchins for behavioral tests of chemical attractiveness. Blank PVC (attractant-free) dispensers were prepared using the same procedure as for the PVC/stimulus mixture and were used as controls. Spirulina was purchased from Livegreen Società Agricola (Oristano, Italy). 

Two PVC/attractant dispensers containing the same microalgae, each inserted into a ceramic filter ring to prevent the dispenser from floating, were positioned in the experimental tank along its outer edge, alternating with two other empty rings (acting as a control), following a radial arrangement [35]. The sea urchin was then placed in the center of the tank, allowing 1 h to respond. The animal movement in the tank was recorded by means of a color digital camera (Samsung SMX-F34, Samsung, Republic of Korea) positioned 60 cm above the tank. The microalgae attractiveness was estimated by taking into account the following measurable parameters: (a) the percentage of tested animals that found the microalgae within 1 h and remained in contact with it for at least 10 min; (b) distance and time (min) traveled (mm) to find it; (c) mean speed (mm/min), determined as the ratio between the distance traveled to reach the microalgae and the time to the target; and (d) tortuosity of the sea urchin’s route to the microalgae substrate, determined as the ratio between the distance (mm) traveled to find the item and the shortest distance (mm) from the center of the tank and the targeted item. Trials were performed on 20 sea urchins for each microalgae species, and each sea urchin was tested with only one chemical at a time.

### 2.6. Data Analysis

Data are expressed as means ± SE. The effects of the different concentrations of the tested compounds on the chemical sensitivity of the sea urchins were evaluated by means of repeated measures ANOVA. For each compound, post hoc comparisons were conducted with Dunnett’s test to assess significant differences between each stimulus concentration and the relative seawater blank control. When data did not conform to a normal distribution (Kolmogorov–Smirnov test for goodness of fit), Friedman’s test was used for comparisons of repeated measures, followed by Dunn’s post hoc test. The attractive effects of Klamath and spirulina on the sea urchins were evaluated by means of an unpaired *t*-test. All statistical analyses were carried out using the Prism program (GraphPad Software, San Diego, CA, USA). Differences were considered significant for *p* < 0.05.

## 3. Results

### 3.1. Chemical Sensitivity to Klamath and Amino Acids

**Figure 1:**
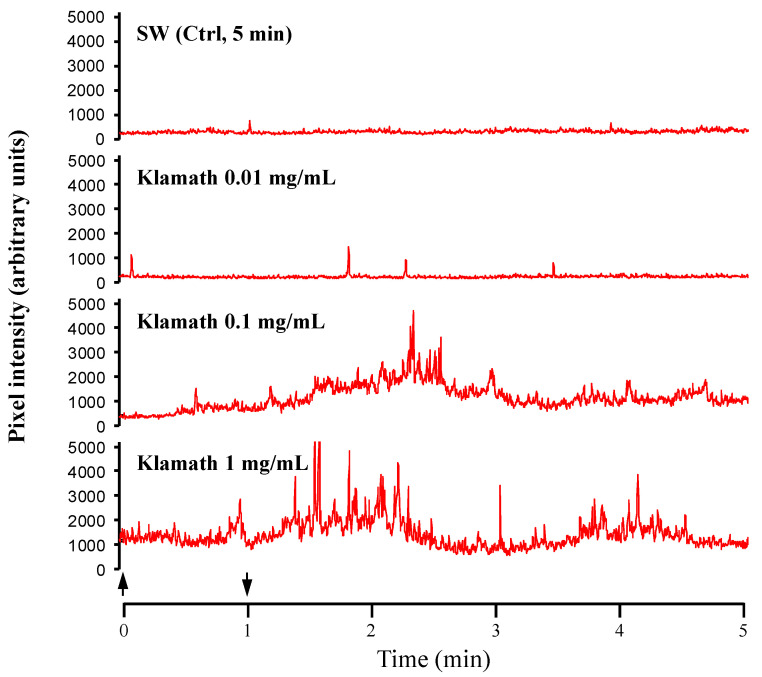
Representative recording of the “urchinogram”, including the sum of all visible movements of spines, pedicellariae, tube feet, and of the whole sea urchin, following supply of the seawater control (SW, CTRL) and of the Klamath microalgae at the doses 0.01, 0.1 and 1 mg/mL. The movement rate was estimated by considering the changes in the mean square difference in pixel intensity between successive frames (analysis performed with the software Aviline at 5 frames/s). Arrows indicate the interval of stimulus delivery.

**Figure 2:**
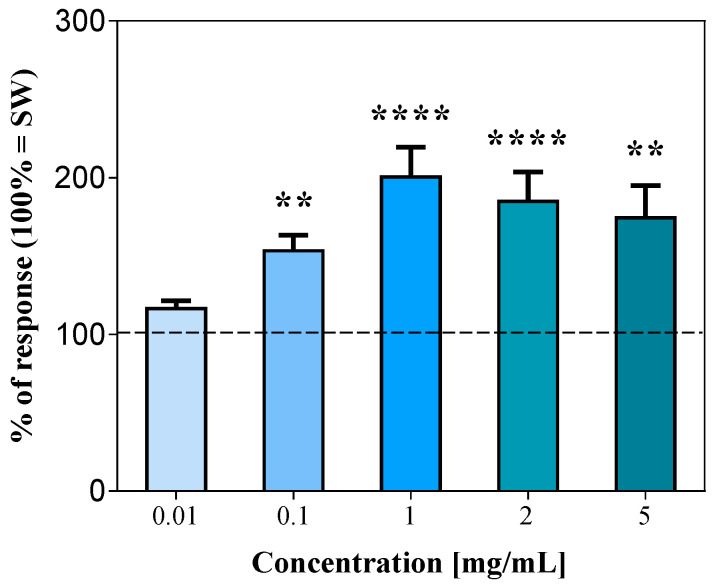
Normalized rate of movements of the sea urchins, expressed as the mean square difference in pixel intensity during a 2 min stimulation ± SE (vertical bars) after supply of the Klamath microalgae, *Aphanizomenon flos-aquae*, compared to seawater (SW = 100% of response, dashed line). ** and **** indicate significant differences for *p* < 0.01 and *p* < 0.0001, respectively (Dunn’s multiple comparison test subsequent to the Friedman Test). Data were obtained from 17 sea urchins.

After acclimatization in the experimental tank, the sea urchins became nearly motionless and, in the absence of chemical stimulation, displayed only negligible, basal activity, which consisted of slow oscillations of a few spines and limited movements of a small number of tube feet. Conversely, when exposed to a stimulating compound, the sea urchins started a stereotyped response that was at first characterized by an increase in the movement rate of the spines combined with a marked enhancement in the tube feet’ projectivity level. Sometimes, this behavior culminated in a fully coordinated locomotory activity of the animal within the experimental tank. The sum of all these responses was considered as an index of the sea urchin’s chemical sensitivity towards a given stimulus, according to Campbell et al. [8]. The Klamath microalgae showed the highest stimulating effectiveness by evoking strong responses in sea urchins. As shown by the urchinogram sample recordings of Figure 1 and histograms of Figure 2, even if ineffective at the lowest tested dose (0.01 mg/mL) with respect to the control with SW (mean square difference in pixel intensity = 101,138 ± 2287, which represents the 100% of the response), starting at 0.1 mg/mL, the microalgae elicited a significant increase in the movement rate of sea urchins to 153.3 ± 9.8% and evoked a peak response up to 200.5 ± 19.1% when tested at 1 mg/mL. The next two doses (2 and 5 mg/mL) of Klamath did not produce any further increase in the sea urchin movement rate (Figure 2).

**Figure 3:**
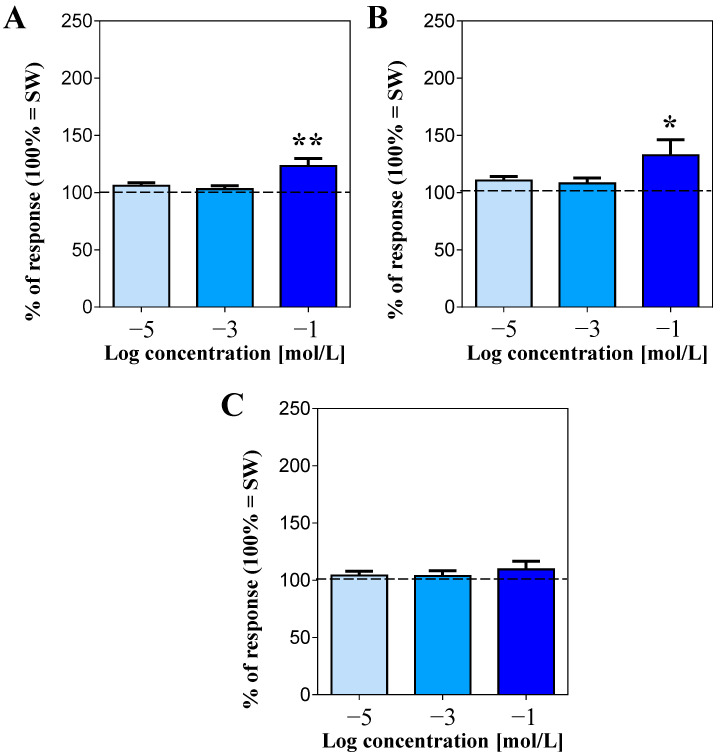
Normalized rate of movements of the sea urchins, expressed as mean square difference in pixel intensity during a 2 min stimulation ± SE (vertical bars) after supply of the essential amino acids leucine (**A**), threonine (**B**), and lysine (**C**), compared to seawater (SW = 100% of response, dashed line). * and ** indicate significant differences for *p* < 0.05 and *p* < 0.01, respectively (Dunnett’s multiple comparison test after one-way ANOVA). Data were recorded from 17 sea urchins for leucine, 16 for threonine, and 15 for lysine.

Among the tested essential amino acids, leucine and threonine were both stimulating with respect to SW, but only at the highest tested concentration (100 mmol/L; Figure 3A and Figure 3B, respectively). In fact, at this concentration, they increased the sea urchin response to 123.3 ± 6.6% and 132.6 ± 13.6%, respectively (mean value of square differences in light intensity for SW was 99,583 ± 1713 in the case of leucine and 95,685 ± 2053 for threonine, in 100% of the responses).

**Figure 4:**
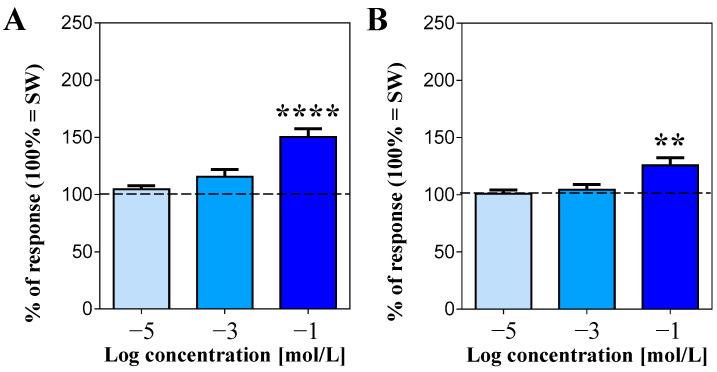
Normalized rate of movements of the sea urchins, expressed as the mean square difference in pixel intensity during a 2 min stimulation ± SE (vertical bars) after supply of the non-essential amino acids arginine (**A**) and proline (**B**), compared to seawater (SW = 100% of response, dashed line). ** and **** indicate significant differences for *p* < 0.01 and *p* < 0.0001, respectively (Dunn’s multiple comparison test after the Friedman Test). Data were recorded from 17 sea urchins for arginine and 16 for proline.

Conversely, no significant changes in the movement rate were detected when the animals were presented with lysine, regardless of the concentration used (mean square difference in pixel intensity for SW = 104,156 ± 2862; Figure 3C). As for the non-essential amino acids tested, both arginine (Figure 4A) and proline (Figure 4B) were stimulating but, similarly to what was observed for leucine and threonine, only at 100 mmol/L, by increasing the sea urchin response to 150.3 ± 7.3% and 125.6 ± 6.7%, respectively (mean square difference in pixel intensity for SW = 98,307 ± 2001 in the case of arginine and 100,958 ± 2773 for proline).

### 3.2. Attractive Effects of Klamath and Spirulina on the Sea Urchins

**Figure 5:**
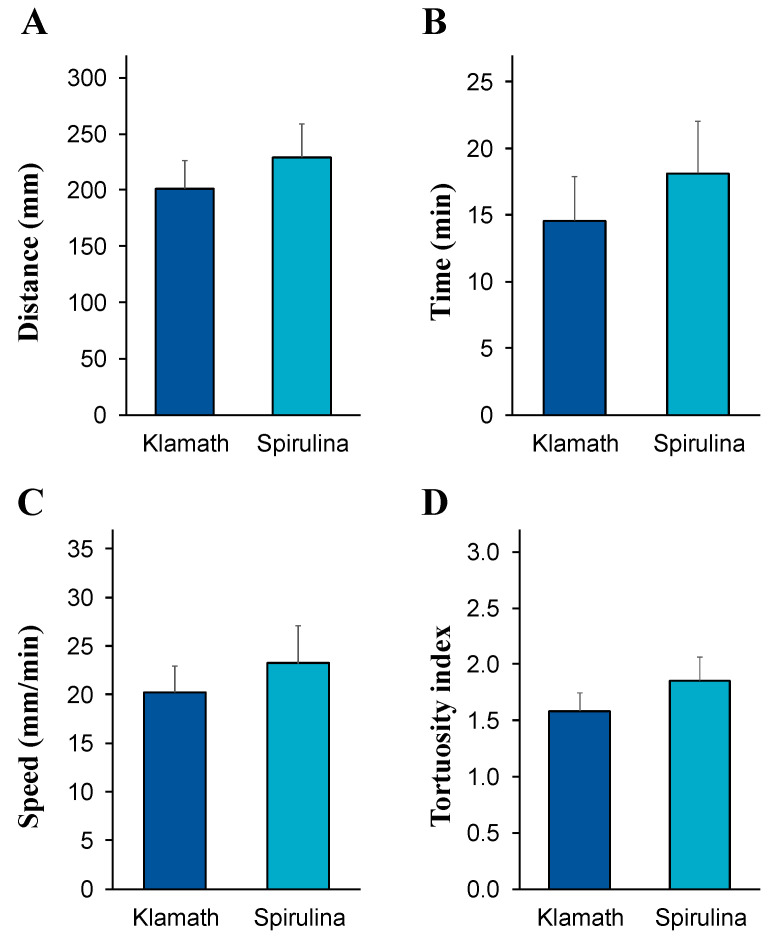
Mean values ± SE (vertical bars) of the distance (**A**), time (**B**), speed (**C**), and tortuosity index (**D**) travelled by the sea urchins to find the microalgae Klamath or spirulina. Data were obtained from 16 and 17 sea urchins for Klamath and spirulina, respectively. No significant differences were found between the two treatments (*p* > 0.05; unpaired *t*-test).

Both microalgae Klamath and spirulina were attractive substrates for the sea urchin *P. lividus*, evoking a positive rheotaxis in most of the tested animals. In the case of Klamath, 16 out of 20 sea urchins (80%) found the algal substrate and remained in contact with it for at least 10 min. Traveling, expressed as a mean distance, was 202.99 ± 23.27 mm (Figure 5A), in a mean time of 14.52 ± 3.10 min (Figure 5B), at a speed of 20.02 ± 2.49 mm/min (Figure 5C) and with a tortuosity index of 1.62 ± 0.15 (Figure 5D). As shown in the same figure, 17 out of 20 tested animals (85%) found the microalgae spirulina, covering a mean distance of 229.31 ± 29.84 mm (Figure 5A), in a mean time of 18.14 ± 3.92 min (Figure 5B), at a speed of 23.21 ± 3.89 mm/min (Figure 5C), displaying a tortuosity index of 1.85 ± 0.21 (Figure 5D). No significant differences were found between the responses of the sea urchins to Klamath and spirulina, regardless of the attractiveness parameter considered (*p* > 0.05; unpaired *t*-test).

## 4. Discussion

The present study shows that the blue-green alga *A. flos-aquae* represents a strong chemical stimulus for the model species *P. lividus*. Using a bioassay based on the “urchinogram”, a method previously used by Solari et al. [45], the Klamath microalgae elicited, within the dose range 0.1–5 mg/L, an overall movement of spines, pedicellariae and tube feet coupled, in some cases, to coordinated locomotion of the animals. All these responses are regarded as classical behavioral indicators of chemical detection in sea urchins [7,8], thus indicating that they are extremely sensitive to Klamath. This fact is not surprising in light of the particularly rich nutritional profile exhibited by this prokaryotic microorganism, which represents a good source of proteins, essential amino acids, polyunsaturated fatty acids, phycocyanins, minerals, carotenoids, and sterols that make it a potential “superfood” [37,38,39,41,46,55]. 

In addition, we found that Klamath, and similarly another blue-green alga, spirulina (*Arthrospira platensis*), displayed a remarkable degree of attractivity towards *P. lividus*. It was previously reported that spirulina is also an effective stimulus for *P. lividus* [45], capable of eliciting strong responses such as the robust activation of both the spine system and tube feet in a fashion similar to what was observed in the present study for Klamath. From a nutritional point of view, spirulina is a well-known source of nutrients including proteins, carbohydrates, essential amino acids, minerals, fatty acids, vitamins, and pigments [56]. Moreover, recent studies have shown several benefits of spirulina-enriched diets for sea urchins such as improvements in gonadic growth and gamete production [44] and the enhancement of contents of compounds showing positive effects in case of degenerative diseases of eggs, such as astaxanthin, a carotenoid with antioxidant properties [57]. Therefore, considering their high nutritional profile, it is not surprising at all that sea urchins display marked sensitivity and rheotaxis towards these microalgae. The benefits of the use of cyanobacteria and microalgae as potential food sources and/or supplements have been known for a long time. For these reasons, they have been extensively cultivated and commercialized in many countries for human consumption [58,59].

In our experiments, sea urchins were also found to be sensitive to a few amino acids from the Klamath biochemical composition. In fact, they responded, even if only at the highest tested concentration, to leucine, threonine, arginine, and proline, thus implying that these amino acids may account, at least in part, for the stimulating effects exerted by the whole microalgae. Sensitivity to amino acids and, in general, to small quantities of nitrogen-containing compounds including amines, nucleotides, and peptides is common in aquatic animals. This is usually regarded as a prerogative of carnivorous predators, as these compounds are prevalent in the tissues of animal prey, while sensitivity to carbohydrates has been more frequently reported in omnivores or herbivores [47,48,60]. Like most other sea urchin species, *P. lividus* is described as a herbivore, but also as an opportunistic, generalist feeder consumer with a preference for seaweed and seagrass [1]. Despite this, only a reduced or partial sensitivity to sugars was previously described for *P. lividus* [45], thus suggesting that sea urchins may preferentially recognize potential feeding substrates which meet at best their nutritional needs by way of the aminoacidic residues. This may also occur in the case of the invertebrate diet counterpart, which is part of the sea urchin diet and is crucial for the assortment of the dietary components ingested [61]. Therefore, such a marked response of the animal to green-blue algae and related compounds might be used for the activation of a stereotyped search strategy aimed at their localization. Further studies are required to investigate whether *P. lividus* shows sensitivity to a potentially larger range of amino acids and especially to other highly nutritional foods like microalgae. It is to be recalled that different sea urchin species could adapt their feeding behavior to the local ecological conditions, therefore developing specific food patterns that may also consist of allochthonous species [62]. However, sea urchins show intraspecific sensitivity due to a plastic rearrangement, which can vary upon changes in the life cycle, size, and age of the animal, and as a result of different availability and distribution of food resources in a specific habitat [1].

In conclusion, the present study reveals the high chemosensitivity of *P. lividus* to Klamath and a few amino acids from its biochemical composition, and a marked rheotaxis to both Klamath and spirulina, although the green-blue algae are not a natural component of the diets of sea urchins. These findings gain further importance considering the commercial potential of echinoderms and their growing importance in aquaculture practice worldwide [63]. Understanding the chemoreceptive features of the sea urchin in the detection of chemicals and discovering key compounds with phagostimulant and/or attractant activity can be essential in formulating feeds or in the supplementation of effective prepared diets suitable for intensive aquaculture systems aimed at limiting diet water soaking [64]. In fact, the preparation of a nutritionally balanced diet can be ineffective if the sea urchin is not able to effectively detect and consume it. Blue-green algae like Klamath and spirulina, which combine high nutritional value with great stimulating and attractive effectiveness, may be good candidates as potential ingredients in the formulation of feed for future aquaculture strategies of these invertebrates. If Klamath and spirulina were included in the prepared diets, they could be used as secondary ingredients in their formulation. Their presence could contribute to increasing the nutritional value of the main ingredients, commonly consisting of other food sources, in particular macroalgae, that are part of the sea urchin’s normal diet. Even if the possibility of toxin contamination (mainly Microcystins) in Klamath has been reported by other authors [65,66], in the present investigation, we excluded such effect as we used a product intended for human consumption, where the occurrence of Microcystins was estimated at ≤1 ppm, which is lower than the recommended limit [67]. 

Nevertheless, further studies are necessary to assess the most favorable dosage at which such algae are detected, as their inclusion in the formulation of supplemented diets can produce important benefits in light of their phagostimulant activity. It could also be useful to investigate whether other non-aminoacidic components of the microalgae composition can trigger a prompt sensory–motor response in sea urchins. Several recent studies on *P. lividus* larvae and adults have focused on improvements in the nutritional content of fresh and formulated diets [25,33,68,69]. A new advance in this field could come from understanding their effectiveness in stimulating food ingestion, helping to improve the palatability of diets consumed by animals.

## Data Availability

Not applicable.

## Supplementary Materials

The following supporting information can be downloaded at: https://www.mdpi.com/article/10.3390/life13071510/s1, Figure S1: experimental layout used to test the responses of sea urchins to waterborne chemical stimuli; Figure S2: recording layout of the sea urchin movements: video photogram showing the experimental tank with the superimposed grid, composed by 13 vertical (a–o) + 9 horizontal (A–I) evenly spaced lines, used to cover the entire area of sea urchin movements; Figure S3: diagram of the study setup: (A) scheme of the experimental arena with the PVC dispenser (grey: Blank PVC (attractant-free) dispensers; green: PVC/stimulus mixture) and the sea urchin positioned in the center of the experimental arena; (B) scheme of an example of the actual (a: dashed black line) and straight (b: red line) trajectories toward the target.
