# Supplementary material for: Blue-Green Algae as Stimulating and Attractive Feeding Substrates for a Mediterranean Commercial Sea Urchin Species, Paracentrotus lividus
Source: Life (Basel). 2023 Jul 5;13(7):1510. doi: 10.3390/life13071510 (PMC10381433; doi:10.3390/life13071510)
Supplement: Supplementary file 1 [file life-13-01510-s001.zip › life-2400372-supplementary.pdf]

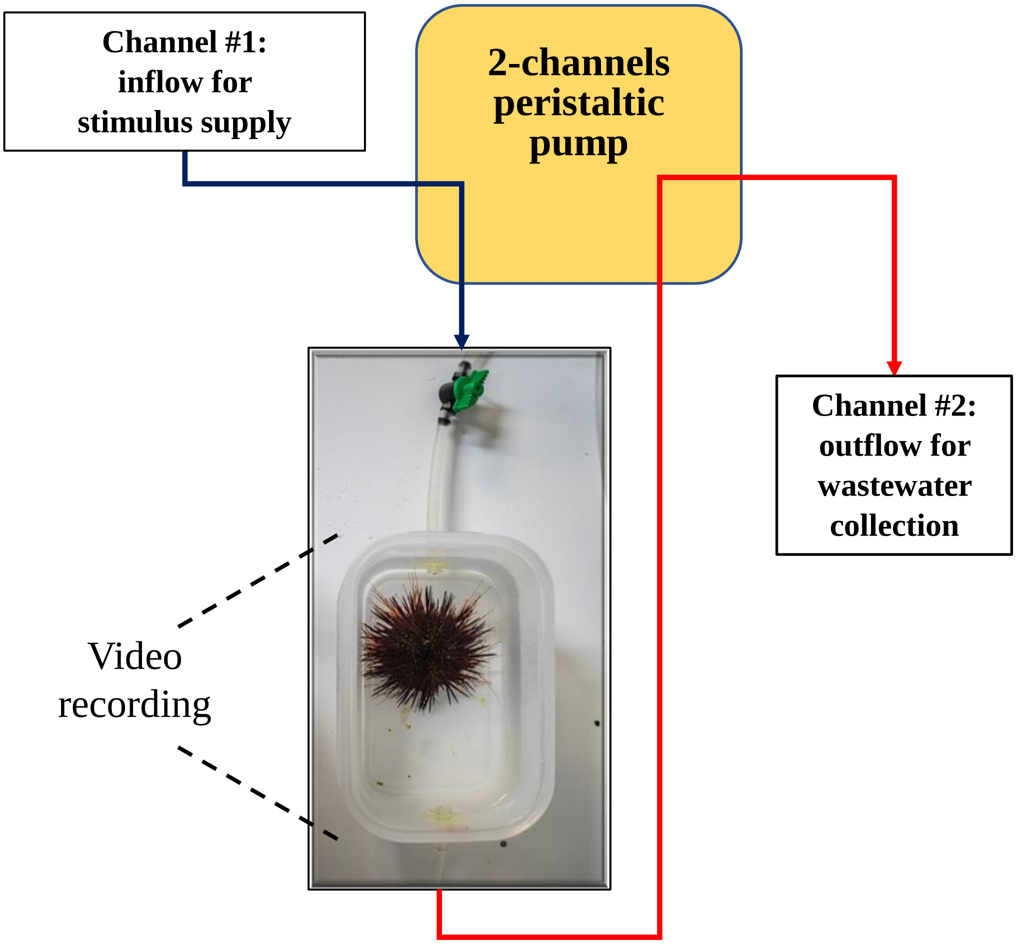

Figure S1. Experimental layout used to test the responses of sea urchins to waterborne chemical stimuli.

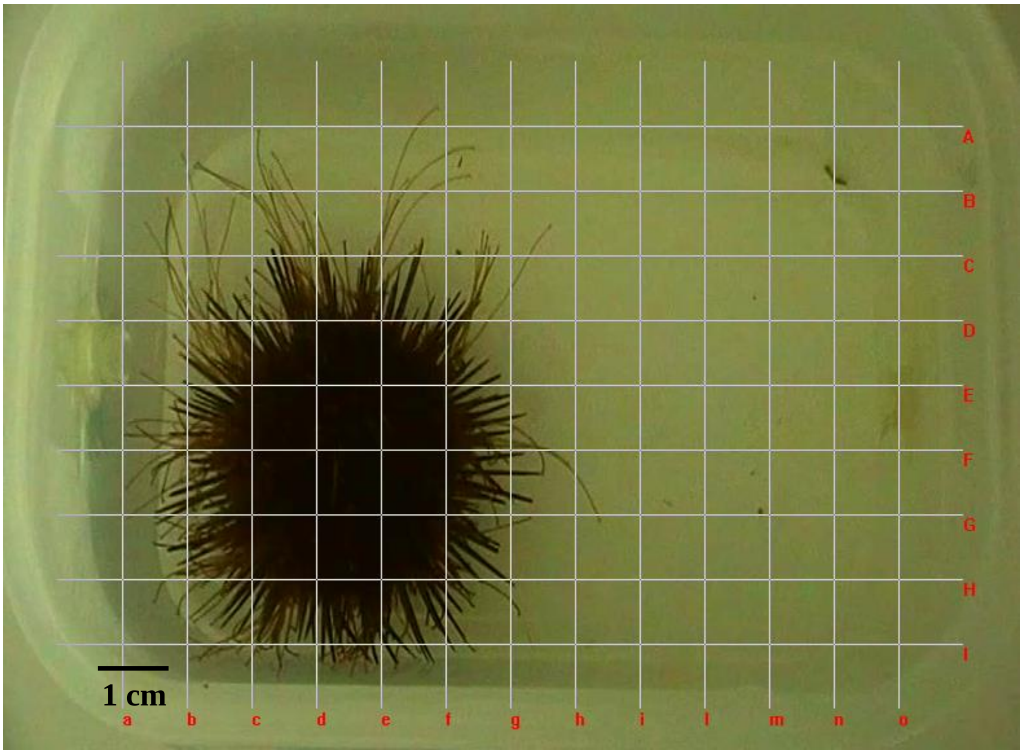

Figure S2. Recording layout of the sea urchin movements: video photogram showing the experimental tank with the superimposed grid, composed by 13 vertical (a–o) + 9 horizontal (A–I) evenly spaced lines, used to cover the entire area of sea urchin movements.

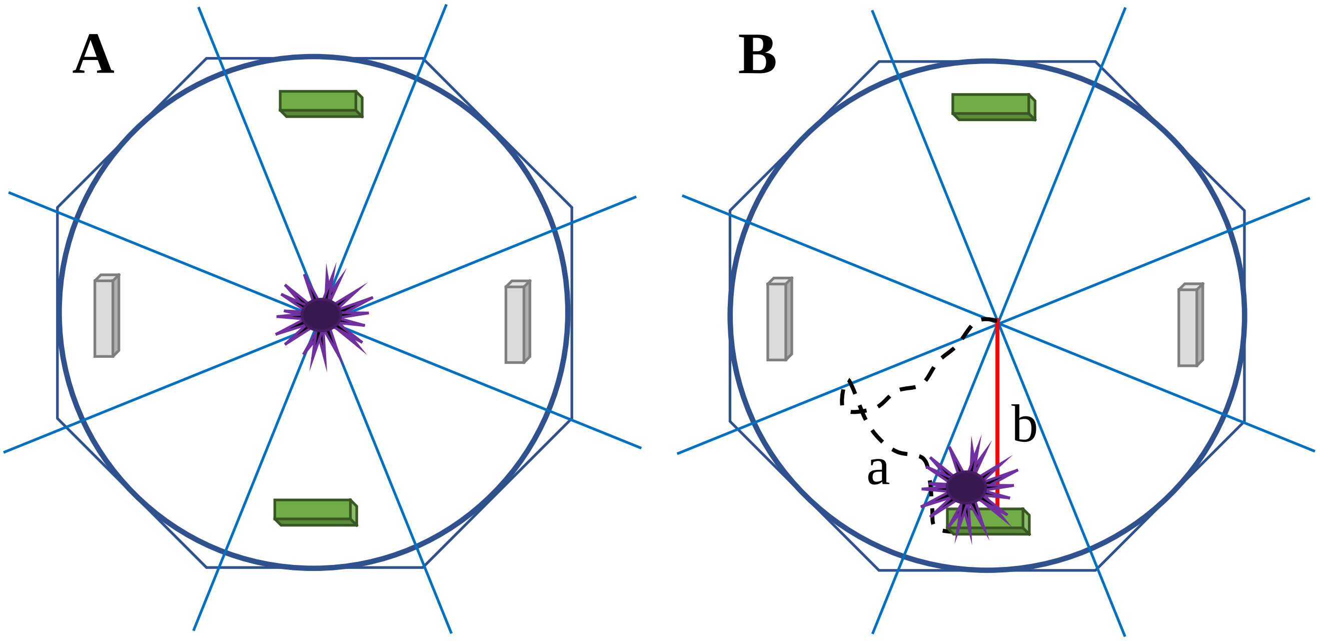

Figure S3. Diagram of the study setup: (A) scheme of the experimental arena with the PVC dispenser (grey: Blank PVC (attractant-free) dispensers; green: PVC/stimulus mixture) and the sea urchin positioned in the center of the experimental arena; (B) scheme of an example of the actual (a: dashed black line) and straight (b: red line) trajectories toward the target.
